# Toward Multi-Parametric Porous Silicon Transducers Based on Covalent Grafting of Graphene Oxide for Biosensing Applications

**DOI:** 10.3389/fchem.2018.00583

**Published:** 2018-11-22

**Authors:** Rosalba Moretta, Monica Terracciano, Principia Dardano, Maurizio Casalino, Luca De Stefano, Chiara Schiattarella, Ilaria Rea

**Affiliations:** ^1^Institute for Microelectronics and Microsystems, Unit of Naples, Naples, Italy; ^2^Department of Chemical Sciences, “Federico II” University of Naples, Naples, Italy; ^3^Department of Physics, “Federico II” University of Naples, Naples, Italy

**Keywords:** porous silicon, graphene oxide, covalent grafting, photoluminescence, optical device

## Abstract

Graphene oxide (GO) is a two-dimensional material with peculiar photoluminescence emission and good dispersion in water, that make it an useful platform for the development of label-free optical biosensors. In this study, a GO-porous silicon (PSi) hybrid device is realized using a covalent chemical approach in order to obtain a stable support for biosensing applications. Protein A, used as bioprobe for biosensing purposes, is covalently linked to the GO, using the functional groups on its surface, by carbodiimide chemistry. Protein A bioconjugation to GO-PSi hybrid device is investigated by atomic force microscopy (AFM), scanning electron microscopy (SEM), water contact angle (WCA) measurements, Fourier transform infrared (FTIR) spectroscopy, steady-state photoluminescence (PL), and fluorescence confocal microscopy. PSi reflectance and GO photoluminescence changes can thus be simultaneously exploited for monitoring biomolecule interactions as in a multi-parametric hybrid biosensing device.

## Introduction

Graphene oxide (GO) is the oxidized counterpart of graphene, characterized by oxygen-bearing functional groups in the form of epoxy, hydroxyl, and carboxyl acids groups on both the basal plane and edges (Dreyer et al., 2010). The oxygen-containing functional groups on the GO sheets make this material more hydrophilic than graphene. In the last decade, GO has attracted great attention because of its unique electronic, mechanical, thermal, and optical properties (Park and Ruoff, 2009; Loh et al., 2010). Moreover, GO can be functionalized with biomolecules without using cross-linkers in aqueous solution, so that this material is particularly interesting for biosensing applications (Jung et al., 2010; Loh et al., 2010; Liu et al., 2012; Zhang et al., 2013).

Several strategies have been published to functionalize GO. In particular, carboxylic acid groups on the GO sheets can be used as reactant sites for immobilization or conjugation of several biological molecules such as proteins, peptides, antibodies, DNA, and so on (Zhang et al., 2010; Wu et al., 2011). Furthermore, GO exhibits steady-state photoluminescence (PL) particular features, such as a broad PL emission from 500 to 900 nm on exposure to near UV radiation, that have been proposed for the development of a new class of optoelectronic devices (Chien et al., 2012). Unfortunately, the PL of a thin layer of GO nanosheets is too weak, mainly due to the oxygen-functional groups producing non-radiative recombination between their electrons and holes present in sp^2^ clusters (Gupta et al., 2014). Oxygen plasma treatment can be used to get higher PL emission from GO (Gokus et al., 2009; Eda et al., 2010). An alternative approach based on the infiltration of GO into large surface area substrate is a valid strategy to enhance the light generation from the resulting composite material, and porous silicon (PSi) is optimal candidate for this task. PSi is a nanostructured material produced by electrochemical anodization of doped crystalline silicon in hydrofluoric acid (HF)-based solution. Pores size and morphology of PSi samples can be properly tuned changing the etching parameters (HF concentration, current density) and the characteristics of the silicon substrate (dopant type, resistivity, crystal orientation). Due to its sponge-like morphology, characterized by a specific surface area of hundreds of m^2^ cm^−3^, PSi is definitely an ideal transducer for the development of several kinds of biosensors (Sailor, 2012; Canham, 2017). In recent papers, hybrid devices constituted by GO electrostatically immobilized on amino-modified mesoporous silicon (i.e., PSi with a pores size <50 nm) were described. In particular, homogeneous monolayer and aperiodic Thue-Morse multi-layered structure made of 64 layers were used in order to infiltrate GO nanosheets by spin-coating. The enhancement and the modulation of the PL signal emitted from GO adsorbed on both the hybrid structures were highlighted, while these phenomena were not observed in the case of GO on crystalline flat silicon (Rea et al., 2014, 2016).

In this work, a chemical procedure to covalently bind GO to PSi surface has been developed in order to realize a stable hybrid device for biosensing purposes. Macroporous silicon, characterized by pores size >50 nm, has been used in infiltrating the GO sheets inside the pores of material. The GO-PSi hybrid device has been covalently conjugated to FITC-labeled protein A (PrA^*^) derived from *Straphilococcus aureus* as a model bioprobe. The effective covalent interaction between GO-PSi and PrA^*^ demonstrates the possibility to realize a robust system for biosensing whose operating mechanism is based on the changes of PSi reflectance and GO photoluminescence.

The development of GO-PSi hybrid device and its interaction with the PrA^*^ have been investigated by Fourier transform infrared spectroscopy (FTIR), spectroscopic reflectometry, steady-state photoluminescence (PL), atomic force microscopy (AFM), scanning electron microscopy (SEM), water contact angle (WCA) measurements, and fluorescence confocal microscopy.

## Materials and methods

### Chemicals

Hydrofluoric acid (HF), undecylenic acid (UDA), N-(3-Dimethylaminopropyl)-N′-ethylcarbodiimide hydrochloride (EDC), N-hydroxysuccinimide (NHS), MES hydrate, tert-Butyloxycarbonyl-NH-PEG-Amine (BOC-NH-PEG-NH_2_), trifluoroacetic acid (TFA), chloroform, tetrahydrofuran, FITC-labeled Protein A (PrA^*^) from *S. aureus* were purchased from Sigma Aldrich (St. Louis, MO, USA). Graphene oxide (GO) nanosheets were purchased from Biotool.com (Houston, TX, USA) as a batch of 2 mg/mL in water with a nominal sheets size between 50 and 200 nm.

### Preparation of graphene oxide

Graphene oxide (GO), 1 mg ml^−1^, was sonicated using an ultrasonic processor for 1 h in ice at 50% of available power amplitude.

### Porous silicon (PSi) layer fabrication and hydrosilylation process

PSi was fabricated by electrochemical etching of *n*-type crystalline silicon (0.01–0.02 Ω cm resistivity, 〈100〉 oriented and 500 μm thick) in HF (5% in weight)/ethanol solution at room temperature (RT). Before the etching process, the silicon substrate was immersed in HF solution for 2 min to remove the oxide native layer. A current density of 20 mA cm^−2^ for 90 s, was applied to obtain a single layer of PSi with a porosity of 61% (n_PSi_ = 1.83 at λ = 1.2 μm), a thickness L of 2.1 μm and a pores dimension between 50 and 250 nm, determined by ellipsometry and SEM imaging (Terracciano et al., 2016). The as-etched PSi was placed in a Schlenk tube containing deoxygenated neat UDA (99% v/v) and allowed to react at 110°C for 18 h under a stream of argon. The treated chip was extensively washed in tetrahydrofuran and chloroform in order to remove the excess of reagent (Shabir et al., 2017).

### Pegylation of PSi layer and covalent grafting of GO

UDA-modified PSi sample was placed in a Schlenk tube containing freshly prepared EDC/NHS aqueous mixture (0.005 M in MES 0.1 M) for 90 min at RT. Sample was rinsed in deionized water three times and dried under nitrogen stream. PEGylation was performed dipping the sample in BOC-NH-PEG-NH_2_ solution (0.4 M, overnight, at 4°C) (Harris et al., 1992; Sam et al., 2010); the excess of reagent was removed rinsing the sample in MES buffer and in deionized water. The *t*-butyloxycarbonyl (BOC) protecting group of amine portion was removed from the PEG covalently bound to PSi surface incubating the sample in a solution of TFA (95% v/v, 90 min, at RT): sample was then washed in deionized water so as to remove the excess of TFA. GO was covalently bound to the PSi surface incubating the sample in sonicated GO (1 mg/ml) in presence of EDC/NHS (0.020 M EDC and 0.016 M NHS in MES 0.1 M, overnight, at RT).

### Covalent grafting of FITC-labeled protein a on PSi layer

GO-modified PSi was incubated in 0.33 mg/ml of FITC-labeled Protein A (PrA^*^) in presence of EDC/NHS (0.020 M EDC and 0.016 M NHS in MES 0.1 M, overnight, at RT). The reaction was conducted over-night at RT.

### Atomic force microscopy

A XE-100 AFM (Park Systems) was used for the imaging of PSi sample before and after functionalization with GO. Surface imaging was obtained in non-contact mode using silicon/aluminum coated cantilevers (PPP-NCHR 10 M; Park Systems) 125 μm long with resonance frequency of 200 to 400 kHz and nominal force constant of 42 N/m. The scan frequency was typically 1 Hz per line. AFM images were analyzed by the program XEI 1.8.1.build214 (Park Systems).

### Scanning electron microscopy

SEM characterization of PSi sample was performed before and after GO functionalization. Images were acquired at 5 kV accelerating voltage and 30 μm wide aperture by a Field Emission Scanning Electron Microscope (Carl Zeiss NTS GmbH 1500 Raith FESEM). InLens detector was used. Samples were tilted at 90° in order to perform SEM analysis in lateral view.

### Water contact angle measurements

Sessile drop technique was used for WCA measurements on a First Ten Angstroms FTA 1000 C Class coupled with drop shape analysis software. Results of WCA are expressed as mean ± standard deviation (s.d.) of at least three measurements on the same sample of three independent experiments (i.e., at least nine measurements for each result).

### Fourier transform infrared spectroscopy

The FTIR spectra of all samples were obtained using a Nicolet Continuμm XL (Thermo Scientific) microscope in the wavenumber region of 4,000–650 cm^−1^ with a resolution of 4 cm^−1^.

### Spectroscopic reflectometry

The reflectivity spectra of PSi sample were measured at normal incidence by means of a Y optical reflection probe (Avantes), connected to a white light source and to an optical spectrum analyser (Ando, AQ6315B). The spectra were collected over the range 600–1,600 nm with a resolution of 1 nm. Reflectivity spectra shown in the work are the average of three measurements.

### Steady-state photoluminescence (PL)

Steady-state photoluminescence (PL) spectra were excited by a continuous wave He-Cd laser at 442 nm (KIMMON Laser System). PL was collected at normal incidence to the surface of samples through a fiber, dispersed in a spectrometer (Princeton Instruments, SpectraPro 300i), and detected using a Peltier cooled charge coupled device (CCD) camera (PIXIS 100F). A long pass filter with a nominal cut-on wavelength of 458 nm was used to remove the laser line at monochromator inlet.

### Laser scanning confocal microscopy

Fluorescent samples were imaged using an inverted fully automated confocal Nikon AR-1 microscope. The NIS elements software was used for image acquisition/elaboration.

## Results and discussion

The structure of GO is characterized by a large amount of hydroxyl, epoxy, and carboxyl groups distributed on the whole surface, which makes this material much more hydrophilic than graphene (Dreyer et al., 2010). GO can be thus processed in aqueous solution in order to directly link biomolecules to its surface for the realization of a biosensor, whose sensing mechanism could be based on changes of GO photoluminescence (Morales-Narváez and Merkoçi, 2012).

**Figure 1:**
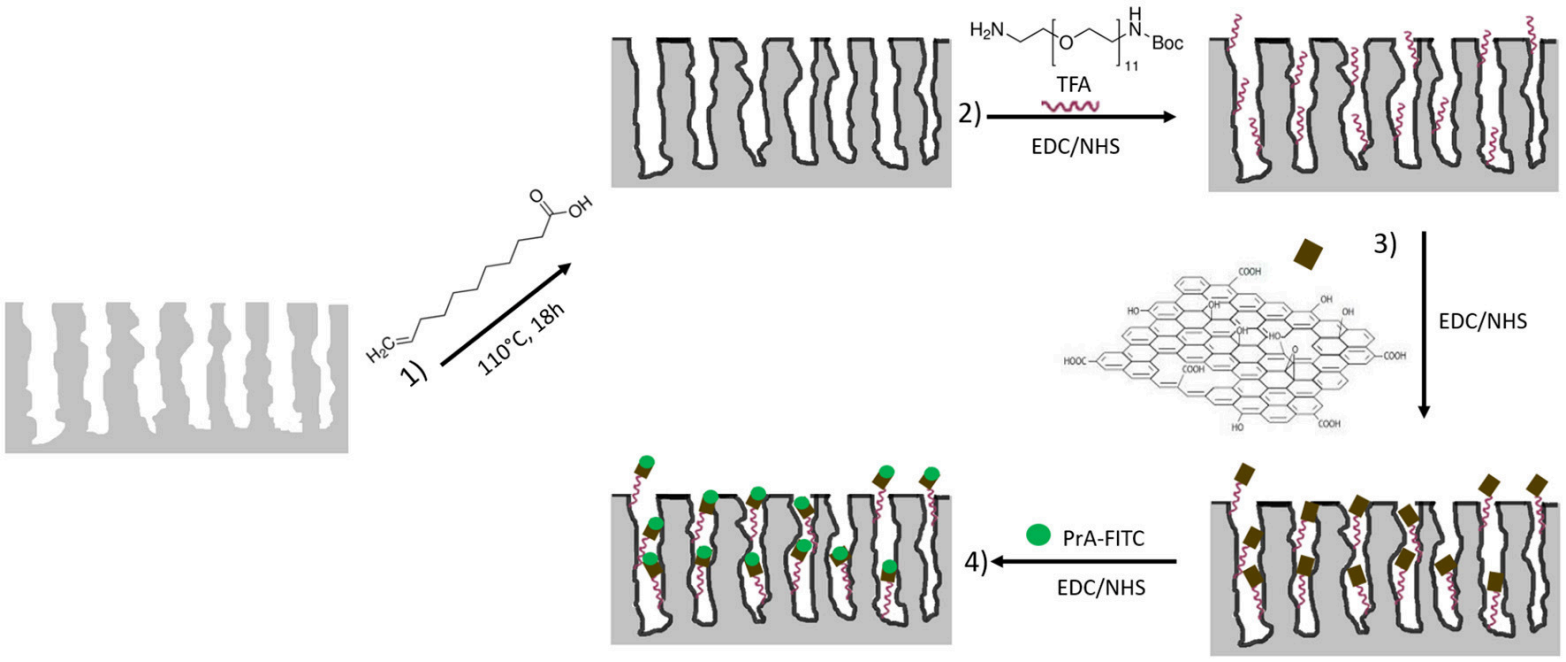
Functionalization scheme of macroporous silicon. **Reaction 1**: hydrosilylation process of PSi using undecylenic acid, 18 h at 110°C. **Reaction 2**: PEGylation process of PSi by EDC/NHS and deprotection of NH-BOC by TFA treatment. **Reaction 3**: immobilization of GO by EDC/NHS on PEGylated PSi. **Reaction 4**: immobilization of PrA^*^ on hybrid device.

The fabrication of a multiparametric hybrid transducer, based on GO covalently immobilized on macroporous PSi surface, required the optimization of GO infiltration, and, before this step, a proper characterization of its behavior in aqueous solutions. GO solubility could be not assured in presence of a biological molecule, so that in Supplementary Information (SI) the interaction of free GO sheets and a FITC labeled PrA^*^, dispersed in demineralized water, is reported. The formation of the complex GO-PrA^*^ was evaluated by DLS (Figure S1), ζ-potential measurements, UV-Vis (Figure S2), and photoluminescence (Figure S3). These results evidenced slight aggregation after GO-PrA^*^ conjugation by EDC/NHS chemistry and a strong interaction between the two systems, which led to changes in UV-Vis absorbance and PL emission. Before infiltration, GO nanosheets had been sonicated until sheets size showed values lower than 100 nm to DLS (data not showed here). After size reduction, GO was covalently bound to the PSi surface following the functionalization scheme reported in Figure 1. Since the main drawback of PSi is its chemical instability in oxidizing environment, such as biological conditions (Ghulinyan et al., 2008), a method to stabilize the surface is mandatory in biosensing applications. A hydrosilylation process has thus been used as a valid strategy to make the PSi a more stable platform. After hydrosilylation, the Si-H surface bonds, typical of as-etched PSi, were converted in Si-C bonds, making the material more robust and resistant to hydrolysis and oxidation (Figure 1, Reaction 1). The thermal reaction between UDA and as-etched PSi induced the formation of an organic monolayer covalently attached to the surface through the formation of Si-C bonds (Boukherroub et al., 2002). The carboxyl acid groups exposed on the surface could be used for further functionalization steps.

The GO grafting to the hydrosilylated-PSi surface required a cross-linker with exposed amino groups. In this study, the PEG molecule was used as bi-functional cross-linker. The covalent grafting of the BOC-NH-PEG-NH_2_ was achieved by carbodiimide chemistry and, after amine group deprotection by acid hydrolysis of BOC with TFA (Figure 1, Reaction 2), the GO sheets were bound on amino-terminal PSi by essentially using the same chemistry (Figure 1, Reaction 3) (Harris et al., 1992). A biosensor transducer always includes a specific bioprobe that recognizes a ligand of interest. Placing a biomolecule into a complex matrix, leaving its properties untouched, requires proper design and stability test of the final hybrid device. In this frame, the interaction between the GO-PSi architecture and a model biological molecule, the Protein A [pure and in its FITC labeled form (PrA^*^)] was studied. The PrA^*^ was covalently bound to the hybrid device using same EDC/NHS chemistry (Figure 1, Reaction 4). In order to verify the effective covalent bond between GO-nanosheets/amino-modified PSi and PrA/GO-modified PSi promoted by carbodiimide chemistry, two negative control samples (NH_2_-PSi_CTR and GO-PSi_CTR) were incubated with GO and PrA (pure and in its FITC labeled form), respectively, without EDC/NHS treatment. Since carbodiimide conjugation should activate carboxyl groups by direct reaction with primary amines via amide bond formation, without EDC/NHS surface treatment, it was not possible to obtain any experimental evidence of covalently bonded GO and PrA on control samples.

**Figure 2:**
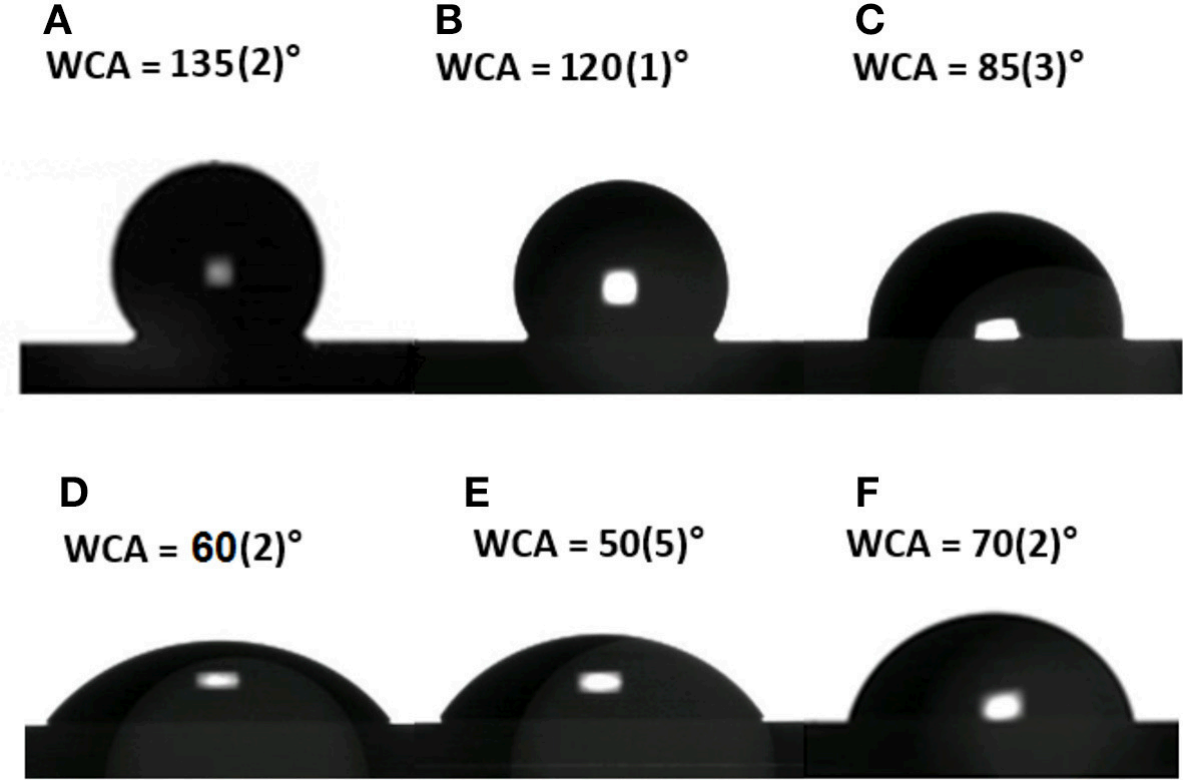
Water contact angle measurement performed on bare PSi **(A)**, after hydrosilylation **(B)**, after PEGylation **(C)**, after TFA treatment **(D)**, after GO functionalization **(E)**, and after PrA^*^ immobilization **(F)**.

Evaluation of surface wettability is a fundamental analysis in the development of such hybrid devices. After each functionalization step, different functional groups were exposed on the GO-PSi chip causing a change in the surface wettability. The as-etched PSi showed a hydrophobic surface quantified by a WCA value of 135 ± 2° (Figure 2A); a weak decrease of wettability value was evaluated after the hydrosilylation process (WCA = 120 ± 1°), mainly due to carboxyl-terminal chain (Figure 2B); the introduction of the hydrophilic group BOC-NH-PEG-NH_2_ induced a further decrease of WCA value to 85 ± 3° (Figure 2C); the removal of the BOC group and the exposure of the hydrophilic amino group, present in the PEG chain, was responsible of a WCA value of 60 ± 2° (Figure 2D); after the GO sheets binding, the presence of the oxygen functional groups made the PSi substrate more hydrophilic with a WCA of 50 ± 5° (Figure 2E); finally, the PrA^*^ bioconjugation on GO-PSi surface increased the wettability of the surface up to a WCA of 70 ± 2° due to the presence of hydrophobic amino acids in the protein structure (Figure 2F). No change of surface wettability was observed in the case of negative control samples (NH_2_-PSi_CTR and GO-PSi_CTR, data not shown).

**Figure 3:**
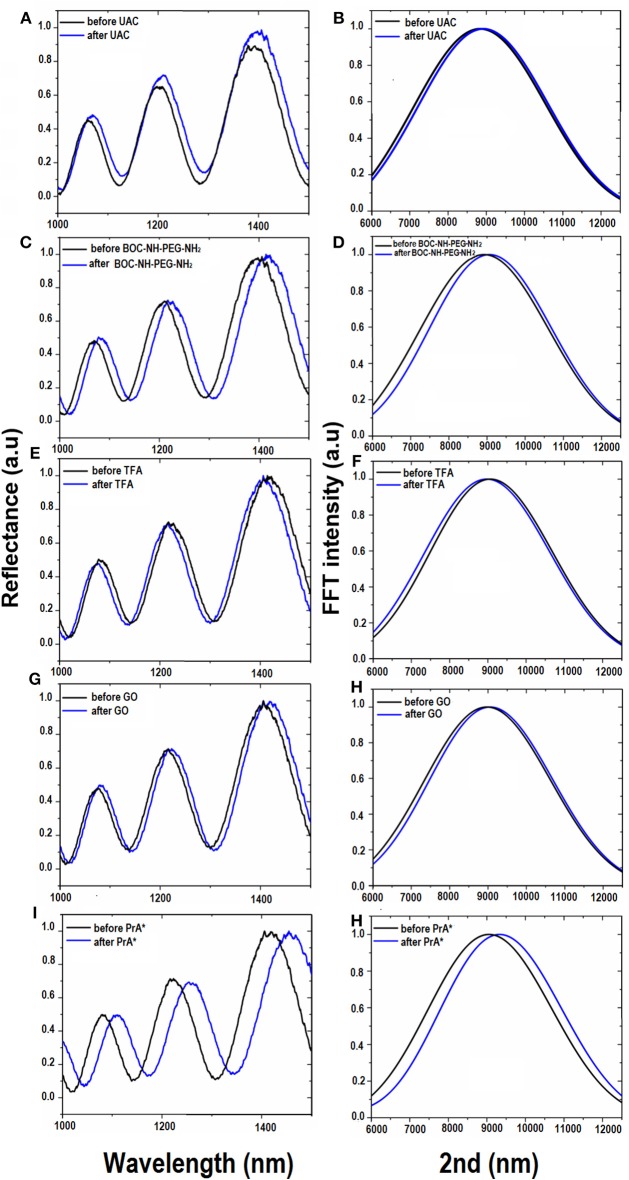
Reflectivity spectra **(A)** and corresponding Fourier transforms **(B)** of PSi before (black line), and after (blue line) UDA treatment. Reflectivity spectra **(C)** and corresponding Fourier transforms **(D)** of UDA-PSi before and after PEGylation with BOC-NH-PEG-NH_2_ (blue line). Reflectivity spectra **(E)** and corresponding Fourier transforms **(F)** of PEGylated PSi before (black line) and after selective deprotection of -NH-BOC by TFA treatment (blue line). Reflectivity spectra **(G)** and corresponding Fourier transforms **(H)** of deprotected PEG-PSi before (black line) and after GO immobilization (blue line). Reflectivity spectra **(I)** and corresponding Fourier transforms **(J)** after PrA^*^ functionalization.

Since chemical functionalization and bioconjugation are additive processes from the material point of view, the optical thickness (i.e., the product of physical thickness, *d*, by the average refractive index *n* of the layer) of the obtained GO-PSi hybrid device, calculated by the FFT of the reflectivity spectrum, was expected to increase. The FFT peak position along the x-axis corresponds to two times the optical thickness (2OT) of the layer (Rea et al., 2014). In Figure 3 are reported the normal incidence reflectivity spectra of PSi before and after hydrosilylation (Figure 3A), and PEGylation process (Figure 3C) together with their corresponding FFTs (Figures 3B,D). Since the physical thickness *d* of PSi layer was fixed, the FFT peak shifts of about 90 nm, after UDA treatment, and of 145 nm after the PEGylation process were due to the increase of the average refractive index of the composite material. This result clearly indicated the two chemical functionalization steps added material layers to the PSi matrix. Figure 3D shows the reflectivity spectrum with the corresponding FFTs (Figure 3E) after the removal of the BOC protector group. The optimization of the reaction was confirmed as a blue shift of the peak of −100 nm (Figure 3F), since the chemical substance has been removed. The GO functionalization and the PrA^*^ conjugation on GO-PSi surface is reported in Figures 3G–J. A FFT peak shift of 90 nm after the PSi surface grafting by GO was an evidence of the occurred functionalization. Finally, the PrA^*^ covalently linked to the hybrid surface induced a further red shift of 275 nm. The analysis of reflectivity spectra of negative control samples (NH_2_-PSi_CTR and GO-PSi_CTR) showed no significant change before and after treatment with GO and PrA solution, respectively, (data not shown).

**Figure 4:**
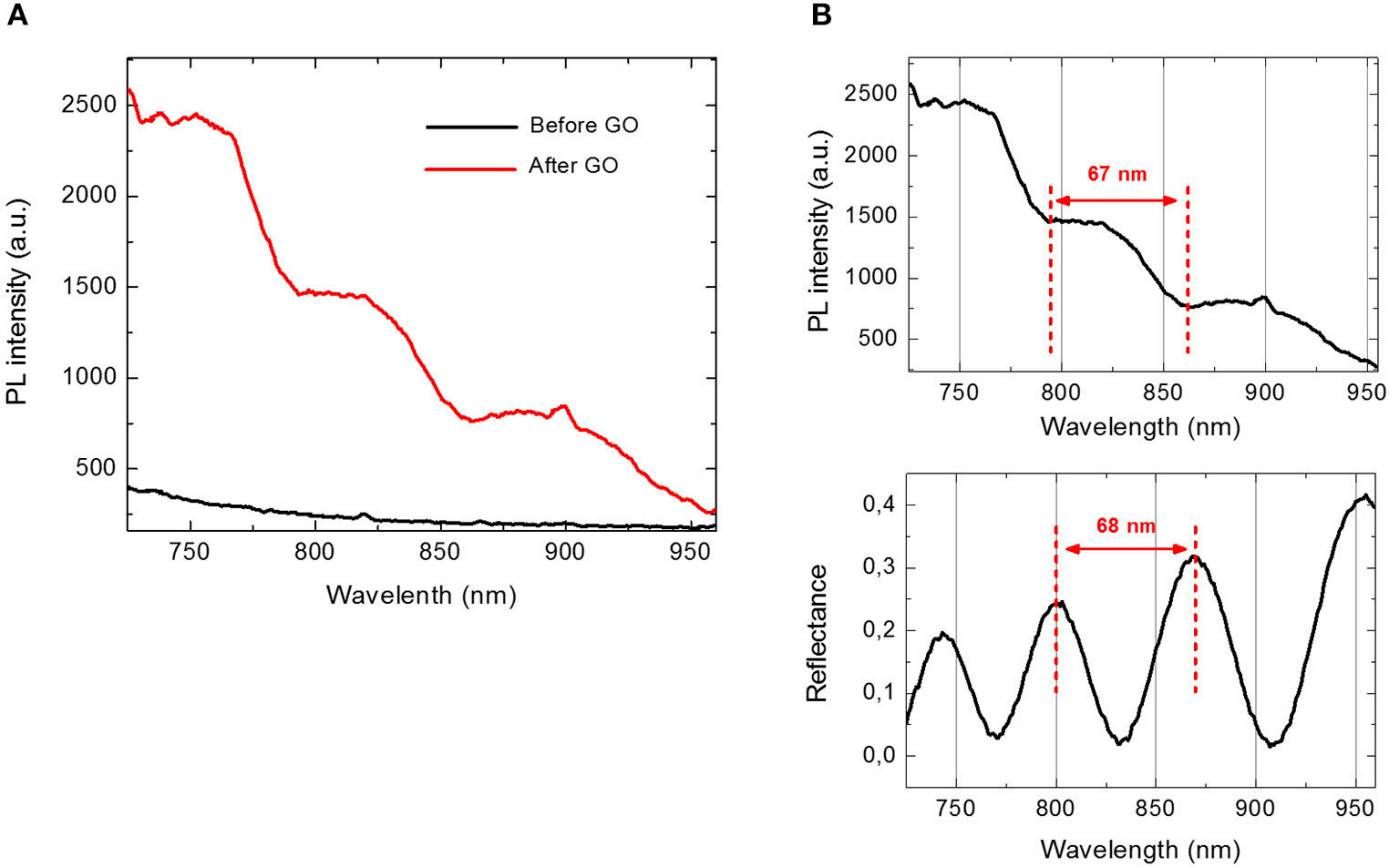
**(A)** Photoluminescence spectra of PSi before (black line) and after GO covalent immobilization (red line) at an excitation wavelength of 442 nm. **(B)** Comparison between photoluminescence spectrum (upper graph) and reflectivity spectrum (lower graph) of GO-PSi device.

The infiltration of GO into PSi was also analyzed by PL measurements. As it can be noted in Figure 4A, in the case of bare PSi, no signal of PL could be detected; on the contrary, the covalent grafting of GO into GO-PSi structure was revealed by a modulation of the PL signal. This was a clear evidence of the GO infiltration into the PSi matrix. The concentration of GO covalently conjugated to PSi was estimated as about 7.5 μg/mL; this value was obtained by comparing the PL spectrum of GO-PSi device with those of some solutions containing different concentrations of GO (Figure S4).

Figure 4B shows a comparison between PL and reflectivity spectra recorded by GO infiltrated in PSi monolayer. The modulation of PL intensity could be explained by considering the optical theory of Fabry-Perot interferometer. Among all the wavelengths, λ_em_ emitted by GO infiltrated in PSi, only those fulfilling the relationship *L* = *m* (λ_em_/2*n*_PSi_), with *L* thickness of the PSi layer and *m* integer, could constructively interfere producing maxima in the PL spectrum of the hybrid structure. The distance between two consecutive photoluminescence maxima was about 67 nm, which well matched the free spectral range of the GO-PSi hybrid structure. In a previous work, we demonstrated that the presence of an interferometer under the GO layer was able to modulate GO photoluminescence (Rea et al., 2014). No change of PL spectra was observed in the case of negative control samples (NH_2_-PSi_CTR and GO-PSi_CTR).

**Figure 5:**
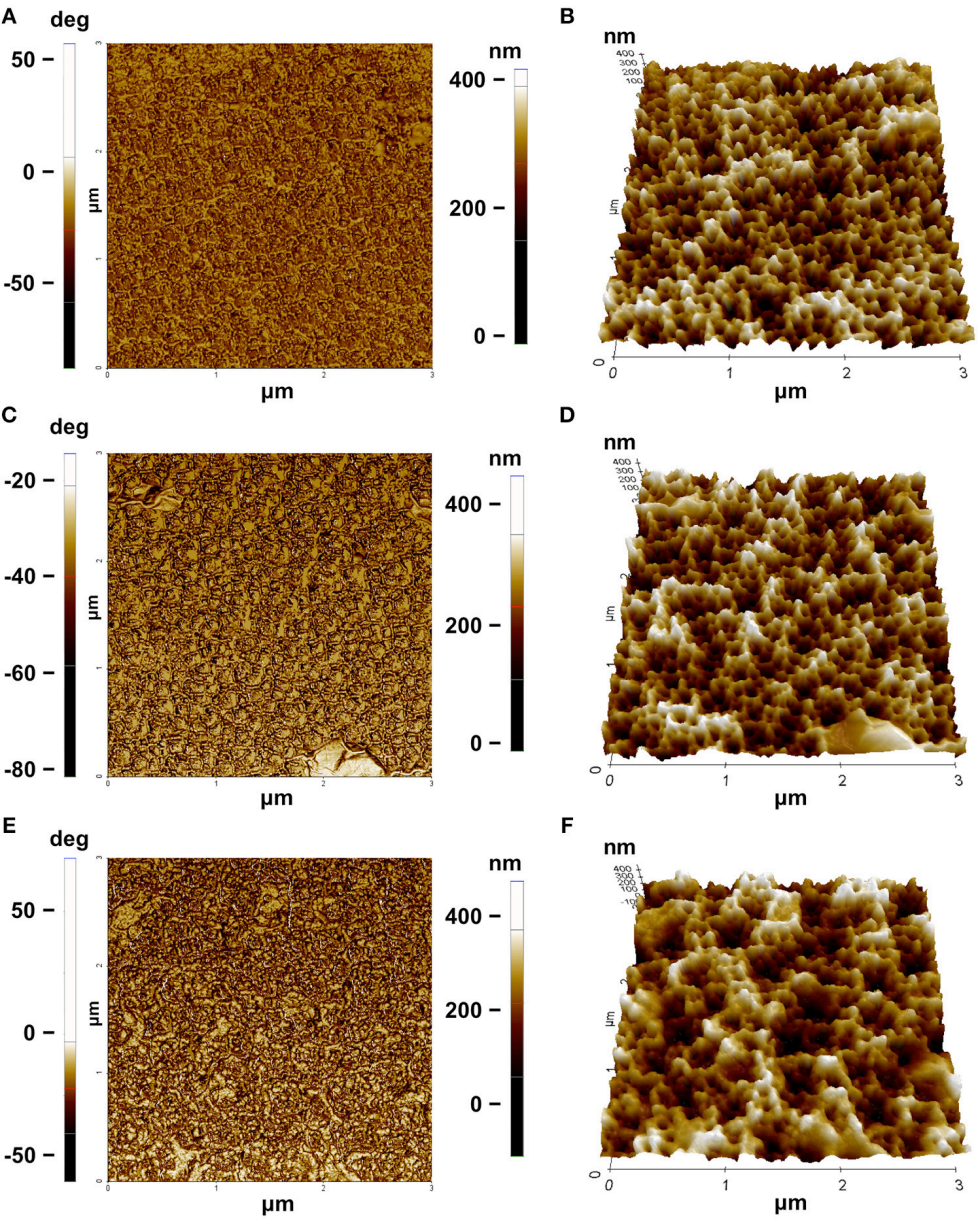
AFM characterization: **(A)** phase and **(B)** three-dimensional rendering of PSi before GO functionalization; **(C)** phase and **(D)** three-dimensional rendering of PSi after GO functionalization; **(E)** phase and **(F)** three-dimensional rendering of PSi after PrA^*^ functionalization.

**Figure 6:**
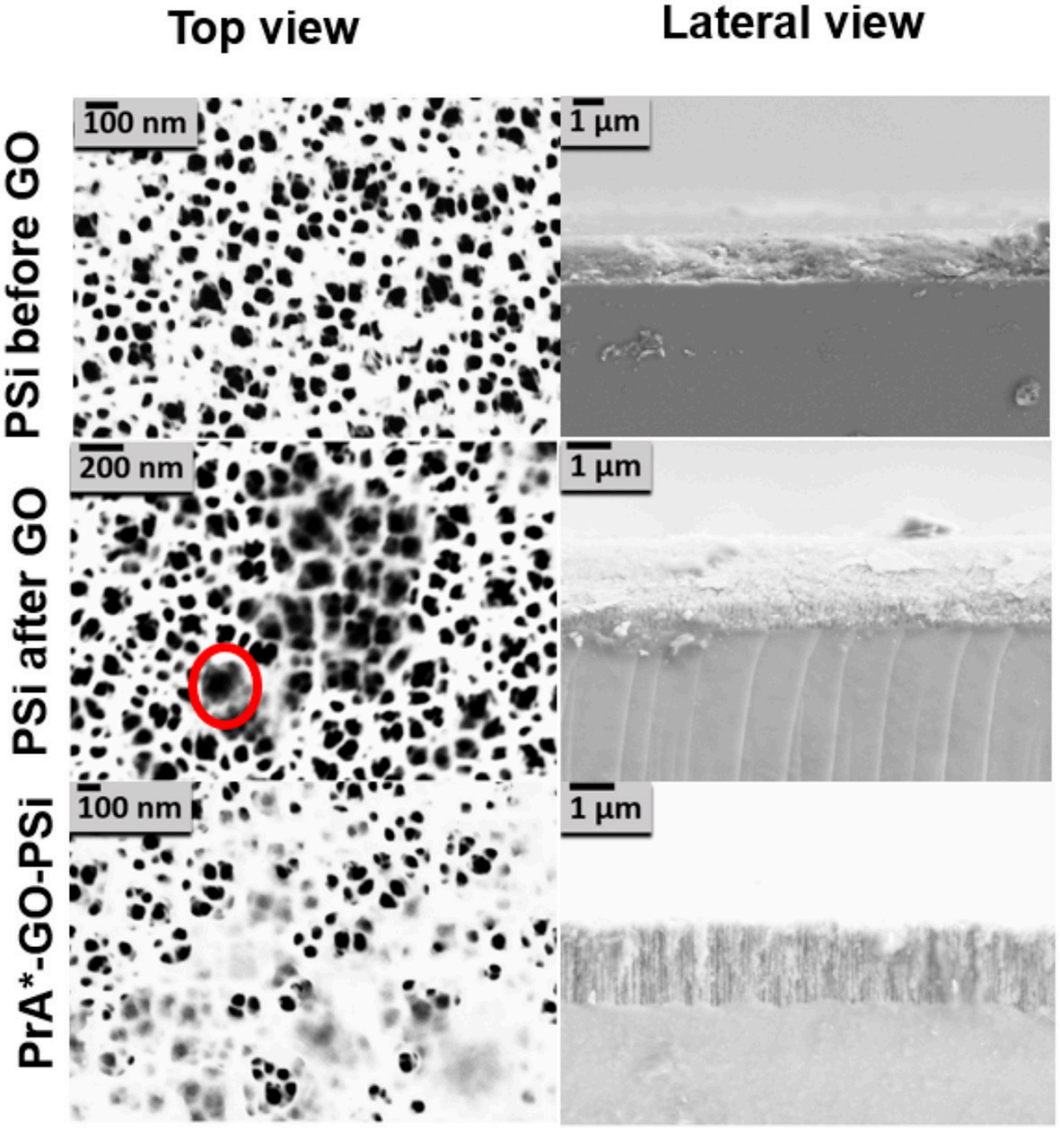
SEM images of PSi before and after GO covalent immobilization, and after PrA functionalization. The red circle indicates the presence of GO.

Morphological features of the surface were highlighted by AFM (Figures 5A–F). The AFM images of bare PSi revealed the presence of hillocks and voids (black zones) of about 100 nm distributed on the whole surface; after the functionalization of the PSi chip with GO, partial pore blocking was evident due to the presence of big GO nano-sheets (white zones) on the PSi surface and some coverage of the surface was visible after the PrA^*^ bioconjugation. The roughnesses of the sample surfaces were calculated by analyzing the AFM images obtaining values of roughness statistical media (Rsm) equal to 0.22 ± 0.01 μm for PSi sample before GO, Rsm = 0.45 ± 0.03 μm after GO, and Rsm = 0.34 ± 0.02 μm after PrA^*^. SEM images of conjugated sample are reported in Figure 6. In the top view, traces of GO (highlighted by red circle) on the PSi surface could be seen, and, in the lateral views, the few GO sheets into the porous matrix were visible. The final PrA^*^-GO-PSi hybrid device had quite almost covered surface and partially blocked pores.

**Figure 7:**
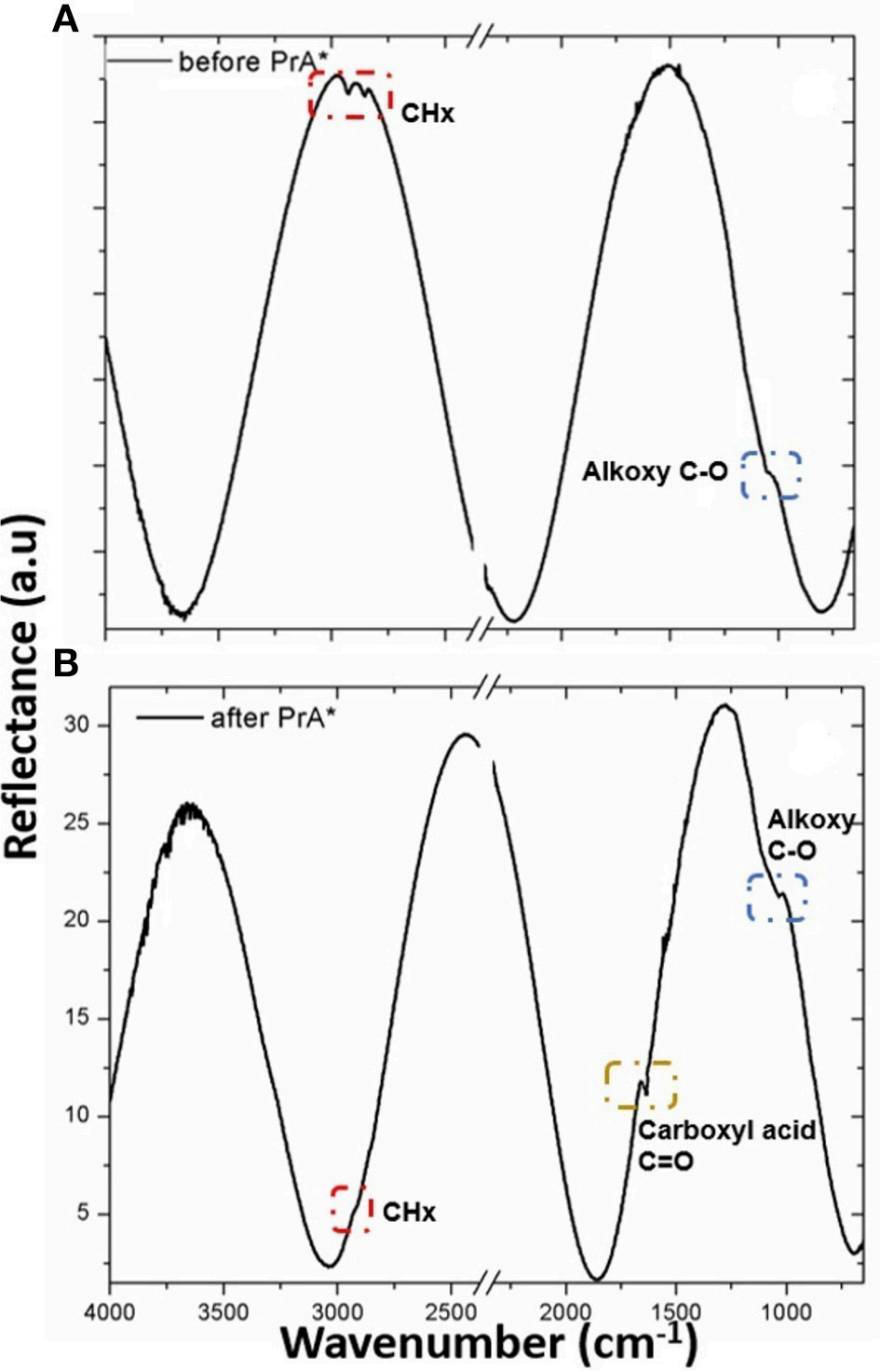
FTIR spectra of GO-PSi hybrid device before **(A)** and after **(B)** PrA^*^ immobilization.

A further analysis of GO-PSi hybrid device functionalization with PrA^*^ was obtained by FTIR spectroscopy (Figure 7). The presence of GO onto hybrid device was confirmed by the presence of CH_x_ at 2,929–2,851 cm^−1^ of carbon networks and the alkoxy C–O at 1,024 cm^−1^ (Yang et al., 2009). After the incubation with the PrA^*^, the hybrid device PrA^*^-GO–PSi showed the stretching bands of CH_x_ at 2,924 cm^−1^ peak, the amide I band at 1,651 cm^−1^ associated with C = O stretching vibration and the alkoxy C–O band at 1,037 cm^−1^ (Socrates, 2007). These data confirmed the covalent bonding of PrA^*^ onto GO-PSi hybrid device.

**Figure 8:**
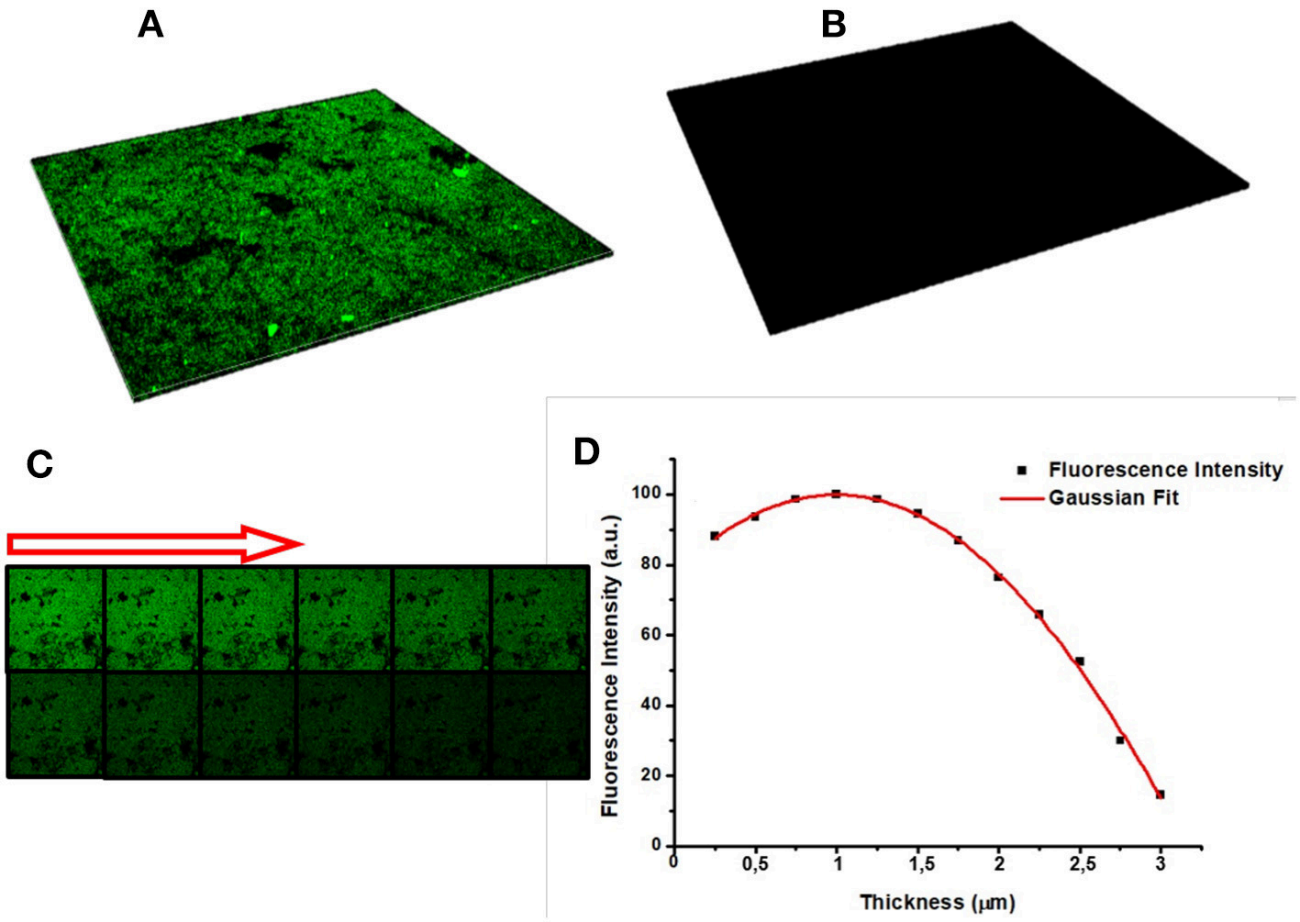
**(A)** Sum of all focal planes of PrA^*^ infiltrated into PSi and **(B)** in the negative control. Sequence of confocal microscope images of protein infiltrated in PSi: the first image is the porous silicon surface, the last is the bottom of the surface, and the arrow indicate the succession **(C)**. Intensity profile of the fluorescence estimated by averaging the intensities of different images concerning the same sample **(D)**.

Confocal microscopy was used for a deeper characterization of the PrA^*^ infiltration process. In particular, Figure 8A is a 3D representation of all focal planes fluorescence recorded by the instrument, confirming the covalent bioconjugation of PrA^*^. In case of the negative control, the corresponding 3D image was completely dark (Figure 8B), and there was not any evidence of aspecific absorption to sample surface. Figure 8C shows the sequence of confocal laser scanning microscope images of the PSi monolayer infiltrated by the PrA^*^: the first image was the one of the top surface while the last was the one recorded at the bottom. Figure 8D quantifies the intensity profiles of the average fluorescence signal and it could be clearly seen that the labeled protein was distributed as a Gaussian function having its maximum value close to the center of the layer. This result further confirm that the protein was penetrated inside the pores (De Stefano and D'Auria, 2007).

## Conclusions

A robust and chemically stable hybrid transducer for biosensing application based on GO, Psi, and PrA^*^, as a model bioprobes, has been designed and demonstrated. EDC/NHS coupling chemistry has efficiently grafted GO to PSi and PrA^*^ to the GO-PSi matrix. Changes in reflectivity optical spectrum and in photoluminescence have been used to characterize the fabrication process but also the transducing features. AFM, SEM, and confocal imaging revealed the main features of the composite structure. The results highlighted promising performances for next generation of multi-parametric biosensors.

## Author contributions

All authors listed have made a substantial, direct and intellectual contribution to the work, and approved it for publication.

### Conflict of interest statement

The authors declare that the research was conducted in the absence of any commercial or financial relationships that could be construed as a potential conflict of interest.

## References

[B1] Boukherroub R. Wojtyk J. T. C. Wayner D. D. M. Lockwood D. J. (2002). Thermal hydrosilylation of undecylenic acid with porous silicon. J. Electrochem. Soc. 149, H59–H63. 10.1149/1.1432679

[B2] Canham L. (2017). Properties of Porous Silicon. Available online at: http://trove.nla.gov.au/work/20545070?selectedversion=NBD13544299 (accessed August 2, 2017).

[B3] Chien C. T. Li S. S. Lai W. J. Yeh Y. C. Chen H. A. Chen I. S. (2012). Tunable Photoluminescence from graphene oxide. Angew. Chemie Int. Ed. 51, 6662–6666. 10.1002/anie.201200474 22623281

[B4] De Stefano L. D'Auria S. (2007). Confocal imaging of protein distributions in porous silicon optical structures. J. Phys. Condens. Matter 19:395009 10.1088/0953-8984/19/39/395009

[B5] Dreyer D. R. Park S. Bielawski C. W. Ruoff R. S. (2010). The chemistry of graphene oxide. Chem. Soc. Rev. 39, 228–240. 10.1039/B917103G 20023850

[B6] Eda G. Lin Y. Y. Mattevi C. Yamaguchi H. Chen H. A. Chen I. S. (2010). Blue photoluminescence from chemically derived graphene oxide. Adv. Mater. 22, 505–509. 10.1002/adma.200901996 20217743

[B7] Ghulinyan M. Gelloz B. Ohta T. Pavesi L. Lockwood D. J. Koshida N. (2008). Stabilized porous silicon optical superlattices with controlled surface passivation. Appl. Phys. Lett. 93:061113 10.1063/1.2969294

[B8] Gokus T. Nair R. R. Bonetti A. Böhmler M. Lombardo A. Novoselov K. S. (2009). Making graphene luminescent by oxygen plasma treatment. ACS Nano 3, 3963–3968. 10.1021/nn9012753 19925014

[B9] Gupta A. Shaw B. K. Saha S. K. (2014). Bright green photoluminescence in aminoazobenzene-functionalized graphene oxide. J. Phys. Chem. C 118, 6972–6979. 10.1021/jp412156x

[B10] Harris J. M. Sedaghat-Herati M. R. Sather P. J. Brooks D. E. Fyles T. M. (1992). “Synthesis of new poly(ethylene glycol) derivatives,” in Poly(Ethylene Glycol) Chemistry, ed J. M. Harris (Boston, MA: Springer US), 371–381.

[B11] Jung J. H. Cheon D. S. Liu F. Lee K. B. Seo T. S. (2010). A graphene oxide based immuno-biosensor for pathogen detection. Angew. Chemie Int. Ed. 49, 5708–5711. 10.1002/anie.201001428 20602383

[B12] Liu Y. Dong X. Chen P. (2012). Biological and chemical sensors based on graphene materials. Chem. Soc. Rev. 41, 2283–2307. 10.1039/C1CS15270J 22143223

[B13] Loh K. P. Bao Q. Eda G. Chhowalla M. (2010). Graphene oxide as a chemically tunable platform for optical applications. Nat. Chem. 2, 1015–1024. 10.1038/nchem.907 21107364

[B14] Morales-Narváez E. Merkoçi A. (2012). Graphene oxide as an optical biosensing platform. Adv. Mater. 24, 3298–3308. 10.1002/adma.201200373 22628274

[B15] Park S. Ruoff R. S. (2009). Chemical methods for the production of graphenes. Nat. Nanotechnol. 4, 217–224. 10.1038/nnano.2009.58 19350030

[B16] Rea I. Casalino M. Terracciano M. Sansone L. Politi J. De Stefano L. (2016). Photoluminescence enhancement of graphene oxide emission by infiltration in an aperiodic porous silicon multilayer. Opt. Exp. 24, 24413–24421. 10.1364/OE.24.024413 27828170

[B17] Rea I. Sansone L. Terracciano M. De Stefano L. Dardano P. Giordano M. (2014). Photoluminescence of graphene oxide infiltrated into mesoporous silicon. J. Phys. Chem. C 118, 27301–27307. 10.1021/jp506539n

[B18] Sailor M. J. (2012). Porous Silicon in Practice: Preparation, Characterization and Applications. San Diego, CA: Wiley-VCH.

[B19] Sam S. Touahir L. Salvador Andresa J. Allongue P. Chazalviel J. N. Gouget-Laemmel A. C. (2010). Semiquantitative study of the EDC/NHS activation of acid terminal groups at modified porous silicon surfaces. Langmuir 26, 809–814. 10.1021/la902220a 19725548

[B20] Shabir Q. Webb K. Nadarassan D. K. Loni A. Canham L. T. Terracciano M. (2017). Quantification and reduction of the residual chemical reactivity of passivated biodegradable porous silicon for drug delivery applications. Silicon 10:349 10.1007/s12633-016-9454-4

[B21] Socrates G. (2007). Infrared and Raman Characteristic Group Frequencies: Tables and Charts. John Wiley & Sons Available online at: https://www.wiley.com/en-us/Infrared+and+Raman+Characteristic+Group+Frequencies%3A+Tables+and+Charts%2C+3rd+Edition-p-9780470093078 (accessed April 14, 2018).

[B22] Terracciano M. De Stefano L. Borbone N. Politi J. Oliviero G. Nici F. (2016). Solid phase synthesis of a thrombin binding aptamer on macroporous silica for label-free optical quantification of thrombin. RSC Adv. 6, 86762–86769. 10.1039/C6RA18401D

[B23] Wu M. Kempaiah R. Huang P. J. Maheshwari V. Liu J. (2011). Adsorption and desorption of DNA on graphene oxide studied by fluorescently labeled oligonucleotides. Langmuir 27, 2731–2738. 10.1021/la1037926 21302946

[B24] Yang X. Zhang X. Ma Y. Huang Y. Wang Y. Chen Y. (2009). Superparamagnetic graphene oxide–Fe_3_O_4_ nanoparticles hybrid for controlled targeted drug carriers. J. Mater. Chem. 19, 2710–2714. 10.1039/b821416f

[B25] Zhang J. Zhang F. Yang H. Huang X. Liu H. Zhang J. (2010). Graphene oxide as a matrix for enzyme immobilization. Langmuir 26, 6083–6085. 10.1021/la904014z 20297789

[B26] Zhang Y. Wu C. Guo S. Zhang J. (2013). Interactions of graphene and graphene oxide with proteins and peptides. Nanotechnol. Rev. 2, 27–45. 10.1515/ntrev-2012-0078

