# Supplementary material for: Toward Multi-Parametric Porous Silicon Transducers Based on Covalent Grafting of Graphene Oxide for Biosensing Applications
Source: Front Chem. 2018 Nov 22;6:583. doi: 10.3389/fchem.2018.00583 (PMC6261979; doi:10.3389/fchem.2018.00583)
Supplement: Supplementary file 1 [file Presentation_1.pdf]

Supporting Information to

Toward multiparametric porous silicon transducers based on covalent grafting of graphene oxide for biosensing applications

Rosalba Moretta[1,2], Monica Terracciano[1], Principia Dardano[1], Maurizio Casalino[1], Luca De Stefano[1*], Chiara Schiattarella[1,3], Ilaria Rea[1]

[1]Institute for Microelectronics and Microsystems, Unit of Naples, Via P. Castellino 111, 80131, Naples, Italy

[2]Department of Chemical Sciences, "Federico II" University of Naples, Via Cynthia, 80126, Naples, Italy

[3]Department of Physics, "Federico II" University of Naples, Via Cynthia, 80126, Naples, Italy

Before covalent functionalization of macroporous silicon (PSi) with graphene oxide (GO), preliminary characterizations and experiments were performed on GO nanosheets in aqueous suspension.

The size of GO sheets was firstly reduced in order to favour the infiltration of GO inside PSi macropores. To this aim, GO was sonicated for 1h at 50% of amplitude and the size distribution of nanosheets (dispersed in water, at a concentration of 1mg/ml) was evaluated by Dinamic Light Scattering (DLS) using a Zetasizer Nano ZS (Malvern Instruments, Malvern, UK) equipped with a He-Ne laser (633nm, fixed scattering angle of 173°, room temperature 25°). The covalent functionalization of GO with biomolecules was also investigated immobilizing on its surface a FTIC labelled-Protein A (PrA*). PrA* was covalently bound through carboxy groups present on GO, using EDC/NHS chemistry. Figure S1 (A) shows the size distributions of GO nanosheets dispersed in water (pH=7) before and after the functionalization with PrA*. Bare material was characterized by two size distributions peaked at 30±10 and 130±70 nm; after functionalization, an increase of the average size to 270±70 nm was observed. The formation of the complex GO-PrA* was also evaluated by ζ-potential measurements. Bare GO showed a ζ-potential of -45±5 mV, while GO-PrA* showed a ζ-potential of -25±5 mV. This variation was ascribed to the positive domains present on the protein surface (Figure S1, B). Figure S2 reports a comparison between the absorbance spectra of GO and GO-PrA*. 2. The UV-vis absorption spectrum of GO was quite different from that reported in the literature. The size reduction of GO nanosheets by sonication could actually partially reduce the GO to rGO (reduced GO), as also reported in literature (Pei, Songfeng, et al., The reduction of graphene oxide, Carbon 50.9 (2012): 3210-3228; Park, Sungjin, et al., Colloidal suspensions of highly reduced graphene oxide in a wide variety of organic solvents,

Nano letters 9.4 (2009): 1593-1597). Anyway, this process poorly affected the reactivity of GO, since some –COOH groups were still available, as demonstrated by formation of GO-PrA* complex in solution. The presence of PrA* in the complex was clearly demonstrated by the contribution at about 500 nm due to the absorption of FTIC fluorochrome. Light emission properties of GO were investigated exciting the nanomaterials at 442 nm. A broad photoluminescence (PL) ranging from 500 nm to 900 nm was observed (Figure S3). After interaction between GO and PrA*, a strong quenching of GO PL was observed; the phenomenon can be ascribed to an electron transfer process from the electronegative GO to the electrophilic PrA*.

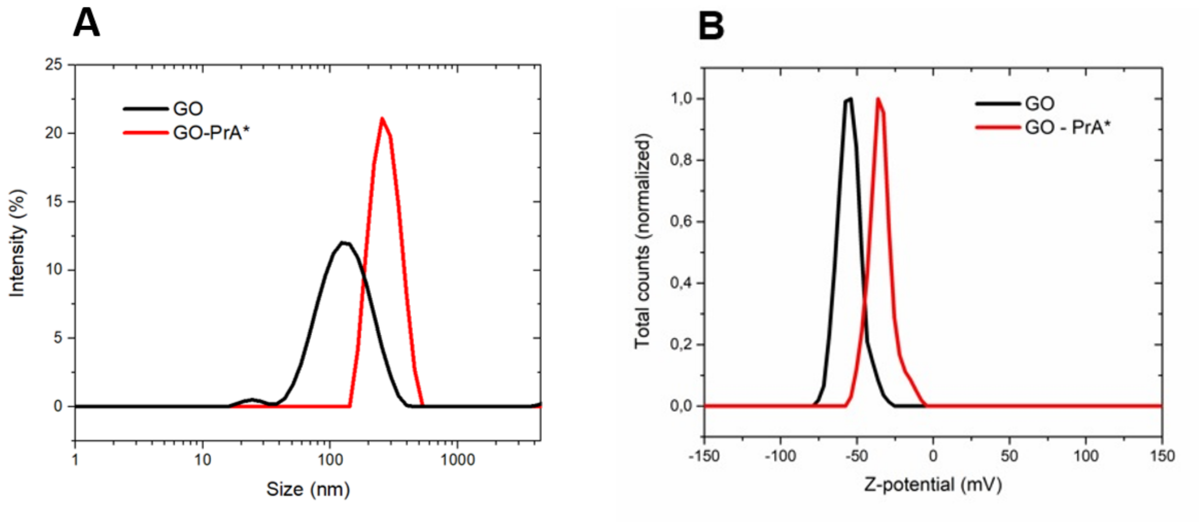

Figure S1. (A) Size distribution of GO (black line) and GO-PrA* (red line) dispersed in water, analysed by DLS. (B) ζ-potential of GO (black line) and GO-PrA* (red line).

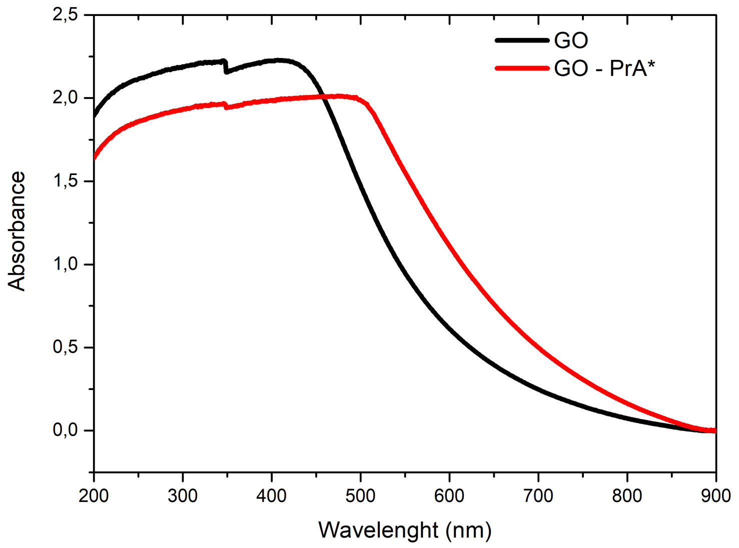

Figure S2. UV-Vis spectra of GO and GO-PrA* dispersed in deionized water.

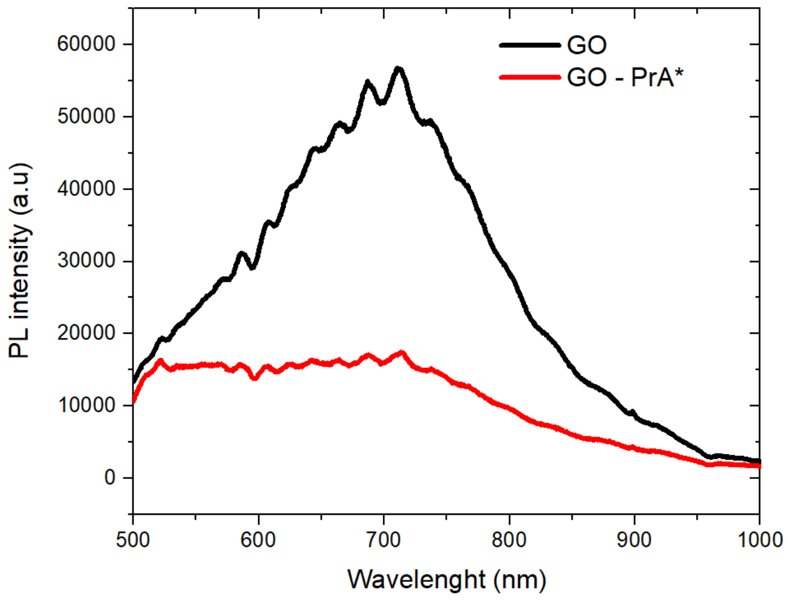

Figure S3. PL spectra of bare GO (black line) and GO-PrA* (red line) at an excitation wavelength of 442 nm.

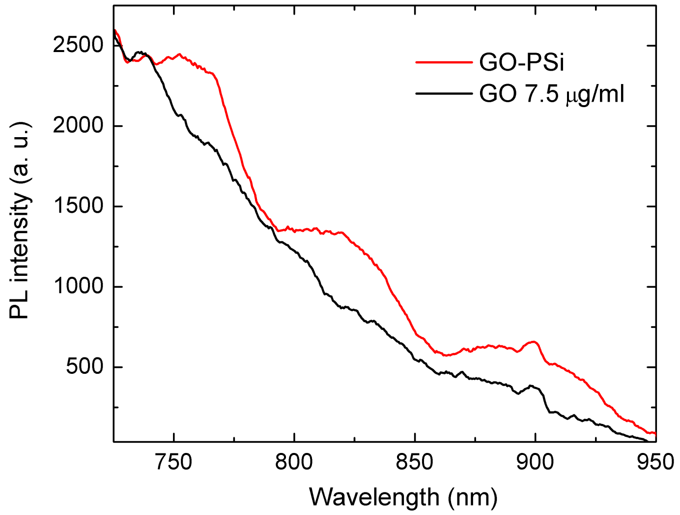

Figure S4. Comparison between PL spectra of GO-PSi device (red curve) and GO in water suspension at a concentration of 7.5 μg/ml (black curve).
